# The gut microbiota regulates bone mass in mice

**DOI:** 10.1002/jbmr.1588

**Published:** 2012-06

**Authors:** Klara Sjögren, Cecilia Engdahl, Petra Henning, Ulf H Lerner, Valentina Tremaroli, Marie K Lagerquist, Fredrik Bäckhed, Claes Ohlsson

**Affiliations:** 1 Centre for Bone and Arthritis Research, Institute of Medicine, Sahlgrenska Academy at University of GothenburgGothenburg, Sweden; 2 Department of Molecular Periodontology, Umeå UniversityUmeå, Sweden; 3 Center for Cardiovascular and Metabolic Research/Wallenberg Laboratory, Sahlgrenska Academy at University of GothenburgGothenburg, Sweden

**Keywords:** GERM FREE, OSTEOPOROSIS, OSTEOIMMUNOLOGY, SEROTONIN, BONE METABOLISM

## Abstract

The gut microbiota modulates host metabolism and development of immune status. Here we show that the gut microbiota is also a major regulator of bone mass in mice. Germ-free (GF) mice exhibit increased bone mass associated with reduced number of osteoclasts per bone surface compared with conventionally raised (CONV-R) mice. Colonization of GF mice with a normal gut microbiota normalizes bone mass. Furthermore, GF mice have decreased frequency of CD4^+^ T cells and CD11b^+^/GR 1 osteoclast precursor cells in bone marrow, which could be normalized by colonization. GF mice exhibited reduced expression of inflammatory cytokines in bone and bone marrow compared with CONV-R mice. In summary, the gut microbiota regulates bone mass in mice, and we provide evidence for a mechanism involving altered immune status in bone and thereby affected osteoclast-mediated bone resorption. Further studies are required to evaluate the gut microbiota as a novel therapeutic target for osteoporosis. © 2012 American Society for Bone and Mineral Research.

## Introduction

The human gut is populated by trillions of bacteria, known as gut microbiota, which collectively contain 150-fold more genes than our human genome.^1^ The gut microbiota is acquired at birth and, although a distinct entity, it has clearly coevolved with the human genome and can be considered a multicellular organ that communicates with and affects its host in numerous ways.^2^ There are several different populations of endocrine cells in the gut that can be affected by the gut microbiota, and hormones released by the bacteria-stimulated endocrine cells can in turn influence host function by entering circulation and by direct communication with terminals of visceral afferent nerves and immune cells.^3^ Accordingly, the gut microbiota has many metabolic functions and contributes to host adiposity.^4^ It is also of importance for maintaining immune homeostasis, ie, protecting the host from invading pathogens while avoiding immune responses to harmless commensal microbes.^5^ Studies have shown that germ-free (GF) animals have immature mucosal immune systems with hypoplastic Peyer's patches containing few germinal centers and reduced number of IgA-producing plasma cells and lamina propria CD4^+^ T cells.^6^ Furthermore, GF mice have reduced number of CD4^+^ T cells in the spleen and fewer and smaller germinal centers within the spleen, suggesting that the gut microbiota is capable of shaping systemic immunity.^7^, ^8^

The skeleton serves as a niche for mesenchymal and hematopoietic progenitors. T-cell precursors generated from hematopoietic stem cells in the bone marrow migrate to the thymus where T-cell development occurs. Mature T cells then recirculate between blood and the secondary lymphoid organs and a large proportion migrate back to the bone marrow where they reside together with other plasma cells.^9^ The bone marrow is a major reservoir for memory B and T cells.^10^ Bone-forming osteoblasts (OB) are derived from pluripotent mesenchymal stromal cells, whereas bone-resorbing osteoclasts (OCL) are derived from hematopoietic stem cells that also generate immune cells. OCLs are specifically derived from the myeloid lineage of hematopoietic cells, and it is the local microenvironment that determines whether the myeloid precursor cell will differentiate into a macrophage, a myeloid dendritic cell, or an OCL.^11^ The presence of macrophage colony-stimulating factor (M-CSF) leads to increased proliferation and survival as well as upregulated expression of receptor activator of nuclear factor-κB (RANK) in precursor cells. This allows RANK ligand (RANKL) to bind and start the signaling cascade that leads to OCL formation.^12^ M-CSF and RANKL are expressed by bone marrow stromal cells as well as by periosteal and endosteal OBs in response to hormones and cytokines stimulating bone resorption.^13^ Tumor necrosis factor (TNF) is an inflammatory cytokine produced by myeloid cells that promotes osteoclastogenesis.^14^ Studies have shown that interleukin 1 (IL-1) is a downstream regulator of the effects of TNFα on inducing osteoclastogenesis and joint damage.^15^, ^16^ The association between inflammation and bone loss is well established, and in autoimmune disease such as arthritis, osteoclastic bone resorption is driven by cytokine-producing activated T cells.^17^, ^18^ Mice lacking T cells (nude mice) have high, normal, or low bone mass depending on the age of the mice when studied.^19^–^21^ Conflicting data exist on whether mice lacking T cells are protected against ovariectomy (ovx)-induced bone loss.^19^, ^21^ To overcome compensatory mechanisms that might be present in mice that lack T cells resulting from a mutation, the effect of ovx in WT mice that were depleted of T cells in vivo by treatment with anti-CD4 and anti-CD8 antibodies were recently investigated. These mice were found to be protected against ovx-induced bone loss, arguing for a role of T cells in this process.^22^

Serotonin (5-hydroxytryptamine, or 5-HT) is a hormone and a neurotransmitter, and the majority of the circulating serotonin is synthesized in the gut by the enterochromaffin cells.^23^ Bone cells express functional serotonin receptors, and gut-derived serotonin has been demonstrated to have negative effects on bone formation in mice.^24^, ^25^ The rate-limiting step in serotonin synthesis in the gut is catalyzed by the enzyme tryptophan hydroxylase-1 (Tph1).^26^, ^27^ The action of serotonin is limited by reuptake into epithelial cells of the intestinal mucosa and serotonergic neurones, where it is broken down.^28^ This uptake is mediated by the serotonin transporter SERT.^29^ Recent studies have reported conflicting data on the effects of serotonin on bone. By treating mice with an inhibitor to Tph1 to decrease gut-derived serotonin synthesis, two studies demonstrated that this led to prevention and treatment of ovx-induced bone loss, whereas one other study showed no effect on bone after similar treatment.^30^–^32^

The importance of the gut microbiota for development of the host's immune system and the suggested connection between gut-derived serotonin and bone led us to investigate the impact of gut microbiota on bone mass. For this purpose, we used the GF mouse as a model.

## Materials and Methods

### Mice

Female GF C57Bl6/J mice were maintained in flexible plastic film isolators under a strict 12-hour light cycle (lights on at 6:00 a.m.). Sterility was routinely confirmed by culturing and PCR analysis from feces using universal bacterial primers amplifying the 16S rRNA gene (8F, AGAGTTTGATCCTGGCTCAG; 338R, TGCTGCCTCCCGTAGGAGT). Age-matched female CONV-R C57Bl6/J mice were transferred to identical isolators at weaning. Both groups of mice were fed an autoclaved chow diet (Labdiet, St. Louis, MO, USA) *ad libitum.* To produce colonized control mice, we colonized 3-week-old GF mice with gut microbiota from C57Bl6/J donor mice as previously described.^4^ Blood was collected from the axillary vein under anesthesia with Ketalar/Domitor vet, and the mice were subsequently killed by cervical dislocation. Tissues for RNA preparation were immediately removed and snap-frozen in liquid nitrogen for later analysis. Bones were excised and fixed in 4% paraformaldehyde or flushed with PBS for isolation of bone marrow cells. The study protocols were approved by the University of Gothenburg Animal Studies Committee.

### Peripheral quantitative computed tomography (pQCT)

Computed tomographic scans were performed with the pQCT XCT RESEARCH M (version 4.5B, Norland, Fort Atkinson, WI, USA) operating at a resolution of 70 µm, as described previously.^33^ Trabecular volumetric bone mineral density (vBMD) was determined ex vivo with a metaphyseal pQCT scan of the proximal tibia. The scan was positioned in the metaphysis at a distance distal from the proximal growth plate corresponding to 3% of the total length of the tibia, and the trabecular bone region was defined as the inner 45% of the total cross-sectional area. Cortical bone parameters were analyzed in the mid-diaphyseal region of the femur.^34^

### MicroCT (µCT)

µCT analyses were performed on the proximal tibia by using Skyscan 1072 scanner (Skyscan N.V., Aartselaar, Belgium), imaged with an X-ray tube voltage of 100 kV and current of 98 µA, with a 1-mm aluminium filter.^35^ The scanning angular rotation was 180° and the angular increment 0.90°. The voxel size was 6.51 µm isotropically. Data sets were reconstructed by using a modified Feldkamp algorithm and segmented into binary images using adaptive local thresholding.^36^, ^37^ Trabecular bone distal of the proximal growth plate was selected for analyses within a conforming volume of interest (cortical bone excluded) commencing at a distance of 338.5 µm from the growth plate, and extending a further longitudinal distance of 488 µm in the proximal direction. Trabecular thickness and separation were calculated by the sphere-fitting local thickness method.^38^ Cortical measurements were performed in the diaphyseal region of femur starting at a distance of 5221 µm from the growth plate and extending a further longitudinal distance of 163 µm in the proximal direction.

### Histomorphometry

Bone histomorphometry was applied to analyze trabecular bone in distal femur. Bones were fixed in 4% paraformaldehyde, dehydrated in 70% EtOH, and embedded in plastic. The trabecular bone was analyzed using longitudinal plastic sections obtained from three standardized sites of marrow cavity with the site distance of 100 µm. These three sites were named as site A, site B, and site C. In each site, the trabecular bone was analyzed in one field. Masson-Goldner Trichrome–stained section with the thickness of 4 µm was used to measure static parameters and unstained section with the thickness of 8 µm to measure dynamic parameters.^39^, ^40^ For the measurement of dynamic parameters, the femora have been labeled with calcein twice, by intraperitoneal injection at eight days, and at one day before sacrifice. The parameters were measured using OsteoMeasure histomorphometry analysis system with software version 2.2 (Osteometrics, Atlanta, GA, USA) and following the guidelines of the American Society for Bone and Mineral Research.^41^

### Blood analysis

Analyses were performed according to the manufacturer's instructions for serum testosterone (RIA, MP Biomedicals/ICN Biomedicals, Costa Mesa, CA, USA), serum calcium (QuantiChrom Calcium Assay Kit [DICA-500], Hayward, CA, USA), serum 25-Hydroxy Vitamin D (EIA, Immunodiagnostic Systems, Herlev, Denmark), Plasma PTH (Mouse Intact PTH ELISA, Immutopics, San Clemente, CA, USA), and serum serotonin (ELISA, IBL-America, Minneapolis, MN, USA). As a marker of bone resorption, serum levels of type I collagen fragments were assessed using a RatLaps ELISA kit (Nordic Bioscience Diagnostics, Herlev, Denmark). Serum levels of osteocalcin, a marker of bone formation, were determined with a mouse osteocalcin immunoradiometric assay kit (Immutopics).

### Quantitative real-time PCR (qRT-PCR) colon

The proximal 2 cm of the colon was used for RNA extraction using the RNeasy Mini Kit (Qiagen, Sollentuna Sweden) with on-column DNase I (Qiagen) digestion according to the manufacturer's instructions. Random hexamer-primed cDNA templates were synthesized from 500 ng of RNAs using the High Capacity cDNA Reverse Transcription Kit (Applied Biosystems, Foster City, CA, USA) according to the manufacturer's instructions. cDNAs were diluted to a final volume of 140 µL, and 2 µL were used for qRT-PCR assays in 25-µL reactions containing 1X SYBR Green Master Mix buffer (Thermo Scientific, Gothenburg, Sweden) and 900 nM gene-specific primers (450 nM primer concentrations were used to assess L32 housekeeping gene expression). A melting curve was performed for each primer pair to identify a temperature where only amplicon, and not primer dimers, accounted for SYBR Green-bound fluorescence. Assays were performed in analytical triplicates using a 7900HT Fast Real-Time PCR System (Applied Biosystems) or CFX96 Real-Time System (Bio-Rad Laboratories, Stockholm, Sweden) and normalized to the level of RNA encoding the L32 ribosomal protein using the ΔΔCT method.^42^ Primer sequences used in this study: Tph1-F, AACAAAGACCATTCCTCCGAAAG; T1-R, TGTAACAGGCTCACATGATTCTC; L32-F, CCTCTGGTGAAGCCCAAGATC; L32-R, TCTGGGTTTCCGCCAGTTT. The primer sequences for Tph1 (ID: 6678411a1) were obtained from the PrimerBank website (http://pga.mgh.harvard.edu/primerbank/). We used a predesigned RT-PCR assay from Applied Biosystems for the analysis of SERT (Mm00439391_m1), IL-6 (Mm00446190_m1), and TNFα (Mm00443258_m1). The mRNA abundance was calculated using the “standard curve method” (User Bulletin 2; PE Applied Biosystems, Carlsbad, CA, USA) and adjusted for the expression of L32 (Mm00777741_sH) ribosomal RNA. RT-PCR analyses for SERT, IL-6, TNFα, and L32 were performed using the ABI Prism 7000 Sequence Detection System (PE Applied Biosystems).

### qRT-PCR femur and bone marrow

Total RNA was prepared from femur and bone marrow using TriZol Reagent (Invitrogen, Lidingö, Sweden). The RNA was reverse transcribed into cDNA using High-Capacity cDNA Reverse Transcription Kit (#4368814, Applied Biosystems, Stockholm, Sweden). RT-PCR analyses were performed using the ABI Prism 7000 Sequence Detection System (PE Applied Biosystems). We used predesigned RT-PCR assays from Applied Biosystems for the analysis of IL-6 (Mm00446190_m1), IL-1 (Mm00434228_m1), and TNFα (Mm00443258_m1) mRNA levels. The mRNA abundance of each gene was calculated using the “standard curve method” (User Bulletin 2; PE Applied Biosystems) and adjusted for the expression of 18S (4308329) ribosomal RNA.

### Flow cytometry

Bone marrow cells were harvested by flushing 5 mL PBS through the bone cavity of one femur using a syringe. After centrifugation at 515*g* for 5 minutes, pelleted cells were resuspended in Tris-buffered 0.83% NH_4_Cl solution (pH 7.29) for 5 minutes to lyse erythrocytes and then washed in PBS. Bone marrow cells were resuspended in RPMI culture medium (PAA Laboratories, Pasching, Austria) before use. The total number of leucocytes in bone marrow was calculated using an automated cell counter (Sysmex, Hamburg, Germany). For flow cytometry analyses, cells were stained with allophycocyanin (APC)-conjugated antibodies to CD4 for detection of T helper cells (Becton Dickinson, Stockholm, Sweden) and fluorescein isothiocyanate (FITC)-conjugated antibodies to CD8 cytotoxic T cells (Becton Dickinson) or Peridinin-chlorophyll proteins (PerCP)-conjugated antibodies to Gr-1/Ly-6G (BioLegend, Cambridge, UK) to eliminate granulocytes and FITC-conjugated antibodies to CD11b for detection of OCL precursor cells (Becton Dickinson). The cells were then subjected to fluorescence activated cell sorter analysis (FACS) on a FACSCalibur (BD Pharmingen, Franklin Lakes, NJ, USA) and analyzed using FlowJo software. Results are expressed as cell frequency (%).

### In vitro culture of osteoclasts from bone marrow cells

Bone marrow cells were flushed from femur and tibias from 8-week-old GF and CONV-R female mice and washed once in α-MEM culture medium (Gibco, Stockholm, Sweden). Bone marrow cells were seeded at 1 × 10^6^ cells/cm^2^ in 96-well plates in α-MEM medium supplemented with 10% fetal bovine serum (Sigma, St. Louis, MO, USA), 2 mM glutamax (Gibco), 50 µg/mL gentamicin (Gibco), 100 U/mL penicillin, 100 µg/mL streptomycin (PEST, Gibco), 30 ng/mL recombinant murine M-CSF (rmM-CSF, cat no. 416-ML; R&D Systems, Minneapolis, MN, USA), and 2 ng/mL recombinant mouse RANKL (cat no. 462-TEC, R&D Systems). After 4 days, with a medium change at day 3, cells were stained for tartrate-resistant acid phosphatase (TRAP, Sigma cat no. 387A). TRAP-positive cells containing three or more nuclei were counted as TRAP + OCL. Because the osteoclasts generated from bone marrow cultures from CONV-R mice were considerably larger and contained more nuclei than osteoclasts from GF mice, we also counted osteoclasts that were spread and contained more than five nuclei.

### Statistical analyses

All the statistical results are presented as the means ± SEM. Between-group differences were calculated using unpaired *t* tests. Comparisons between multiple groups were calculated using a one-way analysis of variance (ANOVA) followed by Tukey's post hoc test. A two-tailed *p* ≤ 0.05 was considered significant.

## Results

### Absence of gut microbiota leads to increased bone mass in mice

**Fig. 1:**
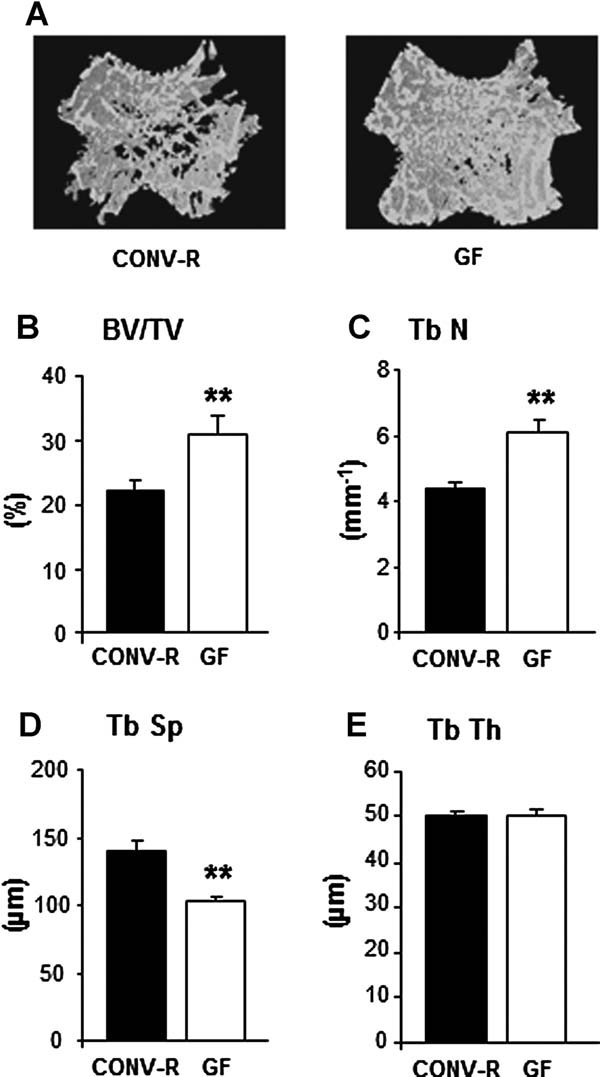
Absence of gut microbiota leads to increased bone mass in mice. Trabecular bone parameters were analyzed by µCT in the distal metaphyseal region of femur from 7-week-old germ-free (GF) and conventionally raised (CONV-R) female mice. (*A*) Representative µCT images of one trabecular section from each group. (*B*) BV/TV (%), trabecular bone volume as a percentage of tissue volume. (*C*) Tb.N (mm^−1^), trabecular number. (*D*) Tb.Sp (µm), trabecular separation. (*E*) Tb.Th (µm), trabecular thickness. Values are given as mean ± SEM, *n* = 8 to 14. *p ≤ 0.05; **p ≤ 0.01 versus CONV-R, Student's *t* test.

To investigate whether the gut microbiota regulates bone mass, we determined the proximal tibial trabecular vBMD in 7-week-old female GF and CONV-R mice. We found that GF mice had increased trabecular vBMD compared with CONV-R mice (277 ± 14 versus 208 ± 10 mg/cm^3^, *p* ≤ 0.01) as determined by pQCT. Further analysis with µCT showed that trabecular bone volume/tissue volume (BV/TV) was increased by 39.4% in the distal femur of GF compared with CONV-R mice (*p* ≤ 0.01, Fig. 1*A, B*). The increased BV/TV was associated with increased trabecular number (+39.5%, *p* ≤ 0.01) and decreased trabecular separation (+26.2%, *p* ≤ 0.01), whereas trabecular thickness was unchanged in GF compared with CONV-R mice (Fig. 1*C–E*). Histomorphometric analysis of the distal femur from 9-week-old mice showed similar results, with increased BV/TV (+39.7%, *p* ≤ 0.01) in the distal metaphyseal region of femur, increased trabecular number (+36.5%, *p* ≤ 0.01), and reduced trabecular separation (+29.2%, *p* ≤ 0.01), whereas trabecular thickness was unchanged in GF compared with CONV-R mice (Supplemental Fig. S1). Histomorphometric analysis showed a decrease in the number of OCLs per bone perimeter, ie, the number of OCLs relative to the external perimeter of the trabecular bone in GF compared with CONV-R mice (–11%, *p* ≤ 0.05, Table 1). Dynamic histomorphometry was used to analyze bone formation, and mineralizing surface per trabecular bone surface was increased in GF compared with CONV-R mice (+17%, *p* ≤ 0.05, Table 1). However, there was no change in mineral apposition rate (Table 1). Analysis of cortical bone in the mid-diaphyseal region of femur by µCT showed that cortical bone area was increased in GF compared with CONV-R mice (CONV-R 0.56 ± 0.01mm^2^; GF 0.61 ± 0.01 mm^2^, *p* ≤ 0.01). We measured serum osteocalcin as a marker of bone formation, and although there was a tendency of increased levels in GF compared with CONV-R mice, it did not reach statistical significance (CONV-R 304.2 ± 29.5 ng/mL; GF 350.4 ± 29.3 ng/mL, *p* = 0.32). As a marker of bone resorption, we measured serum RatLaps (C-terminal telopeptides), and although there was a tendency of decreased levels in GF compared with CONV-R mice, it did not reach statistical significance (CONV-R 38.2 ± 4.8 ng/mL; GF 30.5 ± 3.0 ng/mL, *p* = 0.27).

**Table 1:** Histomorphometric Analysis of Trabecular Bone in the Distal Metaphyseal Region of Femur From 9-Week-Old Germ-Free (GF) and Conventionally Raised (CONV-R) Female Mice

	CONV-R	GF
N.OCL/B.Pm (mm^−1^)	2.92 ± 0.09	2.60 ± 0.11*
MS/BS (%)	27.9 ± 1.3	32.6 ± 0.3*
MAR (µm/day)	1.95 ± 0.15	1.59 ± 0.02
BFR/BS (µm^3^/µm^2^/year)	197 ± 15	190 ± 1

N.OCL/B.Pm = number of OCLs per trabecular bone perimeter; MS/BS = mineralizing surface per trabecular bone surface; MAR = mineral apposition rate; BFR/BS = bone formation rate per trabecular bone surface. Values are given as mean ± SEM; *n* = 5 to 6 for static parameters (N.OCL/B.Pm) and *n* = 3 to 6 for dynamic parameters in each group. Student's *t* test,

* *p* ≤ 0.05 versus CONV-R.

### GF mice have decreased levels of serum serotonin

**Fig. 2:**
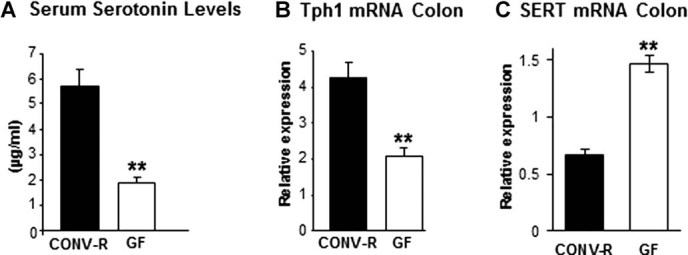
GF mice have decreased peripheral serotonin synthesis. (*A*) Serum serotonin levels in 7-week-old GF and CONV-R female mice. (*B*) qRT-PCR analysis of the expression of tryptophan hydroxylase-1 (Tph1) in proximal colon of 9-week-old GF and CONV-R female mice. (*C*) qRT-PCR analysis of the expression of the serotonin transporter (SERT) in proximal colon of 9-week-old GF and CONV-R female mice. Values are given as mean ± SEM, *n* = 5 to 14. **p ≤ 0.01 versus CONV-R, Student's *t* test.

Gut-derived serotonin has been reported to have negative effects on bone mass, and GF mice had decreased serum serotonin levels compared with CONV-R mice (−67.8%, *p* ≤ 0.01, Fig. 2*A*). We analyzed the mRNA expression of Tph1 and SERT in the proximal colon where bacterial content is high under normal circumstances. In line with the decreased serum serotonin levels, we found decreased mRNA expression of Tph1 (−50%, *p* ≤ 0.01, Fig. 2*B*) and increased mRNA expression of SERT (122%, *p* ≤ 0.01, Fig. 2*C*) in the proximal colon of GF compared with CONV-R mice.

Sex steroids are potent regulators of bone mass, and to assess if the gut microbiota reduced the bone mass indirectly by modulating serum levels of sex steroids, we determined the testosterone levels, which were undetectable in both GF and CONV-R female mice. Because it is difficult to obtain reliable measurements of serum estradiol levels in mice, we used the uterus weight as an indicator of estrogen status, which did not differ (CONV-R 80.3 ± 8.6 mg; GF 87.1 ± 16.4 mg).

### Calcium homeostasis in GF mice

To investigate if calcium homeostasis was affected in GF mice, we measured serum calcium (Ca^2+^), plasma parathyroid hormone (PTH) and serum 25-hydroxy vitamin D (25[OH]D_3_) levels. No significant differences were observed between the two groups (Supplemental Table S1).

### GF mice exhibit an altered immune status in bone

**Fig. 3:**
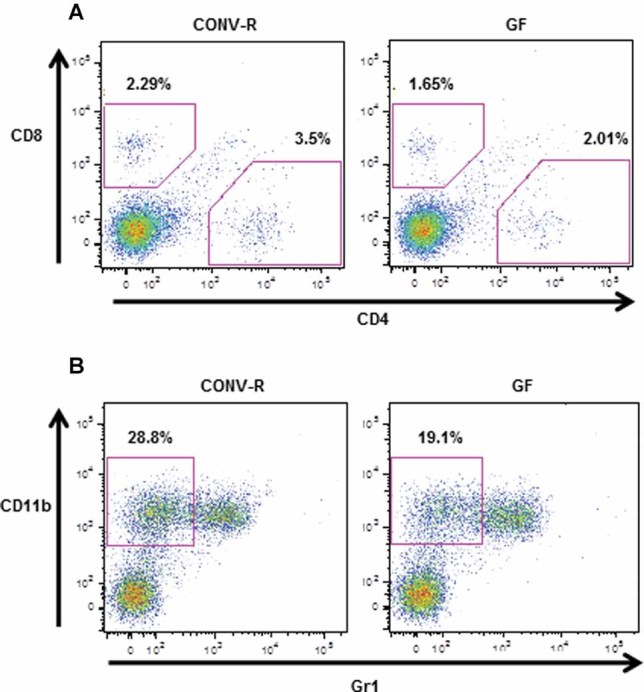
GF mice have a decreased frequency of CD4 T cells and osteoclast (OCL) precursor cells (CD11b^+^/Gr1^–^) in bone marrow. Femur bone marrow cells from 7-week-old GF and CONV-R female mice were stained with antibodies recognizing CD8, CD4, CD11b, and Gr1 and analyzed by flow cytometry, gating as shown. (*A*) Values represent the percentage of CD8^+^ and CD4^+^ cells in the total bone marrow population. Data are representative of two independent experiments. (*B*) Values represent the percentage of CD11b^+^/Gr1^–^ cells (OCL precursor cells; see text for details) in the total bone marrow population. Data are representative of two independent experiments.

**Fig. 4:**
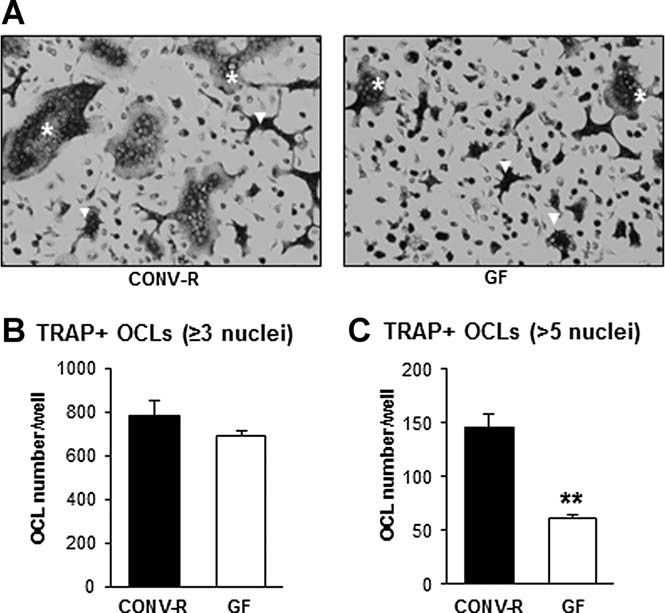
Osteoclast formation in vitro is decreased in bone marrow cultures from GF mice. Bone marrow cells were flushed from femur and tibias from 8-week-old GF and CONV-R female mice, cultured for 4 days, and then stained for tartrate-resistant acid phosphatase (TRAP). (*A*) Representative images showing TRAP + OCLs in culture from each group. White stars (*) indicate examples of spread OCLs with more than five nuclei, and white arrows (Δ) indicate examples of OCLs with more than three but less than five nuclei. (*B*) Quantitative data for TRAP-positive cells containing three or more nuclei. (*C*) Quantitative data for spread TRAP-positive cells containing more than five nuclei. Values are given as mean ± SEM, *n* = 4 to 5. **p < 0.01 versus CONV-R, Student's *t* test.

**Fig. 5:**
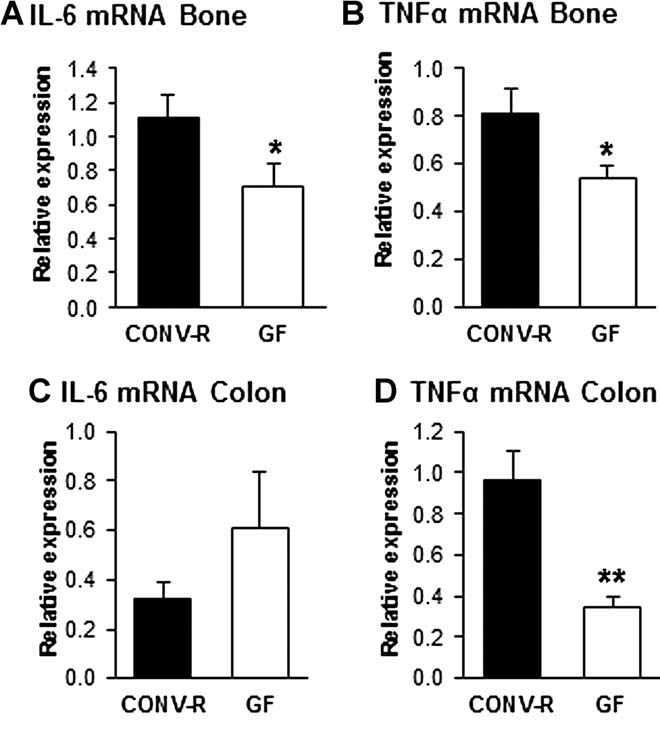
GF mice have decreased expression of IL-6 and TNFα in bone. QRT-PCR analysis of the expression of (*A*) interleukin-6 (IL-6) and (*B*) tumor necrosis factor alpha (TNFα) in tibias from 7-week-old GF and CONV-R female mice and (*C*) IL-6 and (*D*) TNFα in proximal colon of 9-week-old GF and CONV-R female mice. Values are given as mean ± SEM, *n* = 5 to 14. *p ≤ 0.05 versus CONV-R, Student's *t* test.

The close connection between the immune and the skeletal system prompted us to investigate possible immune effects in GF mice that could have an impact on bone mass. FACS analysis of bone marrow showed similar number of bone marrow cells in femur in GF and CONV-R mice (CONV-R 11.7 ± 0.4 × 10^6^; GF 11.0 ± 0.6 × 10^6^, *n* = 8–14, NS) but a decreased frequency of CD4^+^ T cells (CONV-R, 100 ± 4%; GF 75 ± 6%, *n* = 8–14, *p* ≤ 0.01, Fig. 3*A*) and no difference of CD8^+^ T cells (CONV-R 100 ± 7%; GF 95 ± 7%, *n* = 8–14, NS, Fig. 3*A*). CD11b protein is expressed on the surface of various myeloid lineage cells, especially on granulocytes, which constitute a major part of the cells in bone marrow in mice. CD11b-positive cells that are Gr1 negative (to eliminate granulocytes) have the potential to differentiate into OCLs in vitro and are referred to as OCL precursor cells.^43^ The frequency of OCL precursor (CD11b^+^/Gr1^–^) cells in bone marrow was decreased in GF compared with CONV-R mice (CONV-R 100 ± 4%; GF 84 ± 2%, *n* = 8–14, *p* ≤ 0.01, Fig. 3*B*). To assess if the decreased frequency of CD11b^+^/Gr1^–^ cells in bone marrow gave rise to fewer OCLs, we cultured bone marrow cells in vitro in the presence of M-CSF and RANKL and stained for TRAP (Fig. 4*A*). TRAP^+^ OCLs containing three or more nuclei were decreased by 12.3% in GF compared with CONV-R mice, although this did not reach statistical significance (Fig. 4*B*). Because the OCLs generated from bone marrow cultures from CONV-R mice were considerably larger and contained more nuclei than osteoclasts from GF mice, we also counted TRAP^+^ OCLs that were spread and contained more than five nuclei. These were decreased by 57.8% (*p* < 0.01) in GF compared with CONV-R mice (Fig. 4*C*). One of the mechanisms for the effects of the immune system on bone is via altered mRNA expression of cytokines by immune cells. mRNA expression analysis demonstrated decreased expression of IL-6 and TNFα in bone (IL-6–37%, *p* ≤ 0.05; TNFα –34%, *p* ≤ 0.05; Fig. 5*A, B*) of GF compared with CONV-R mice. We then investigated mRNA expression of these cytokines in gut, and the expression of TNFα but not IL-6 was decreased (–64%, *p* ≤ 0.01, Fig. 5*C, D*) in colon of GF compared with CONV-R mice. mRNA expression analysis of TNFα, IL-6, and IL-1, a downstream regulator of the effects of TNFα, in bone marrow showed decreased expression of TNFα and IL-1 but increased IL-6 expression in GF compared with CONV-R mice (Supplemental Fig. S2).

### Colonization of GF mice with a normal gut microbiota normalizes bone mass

**Fig. 6:**
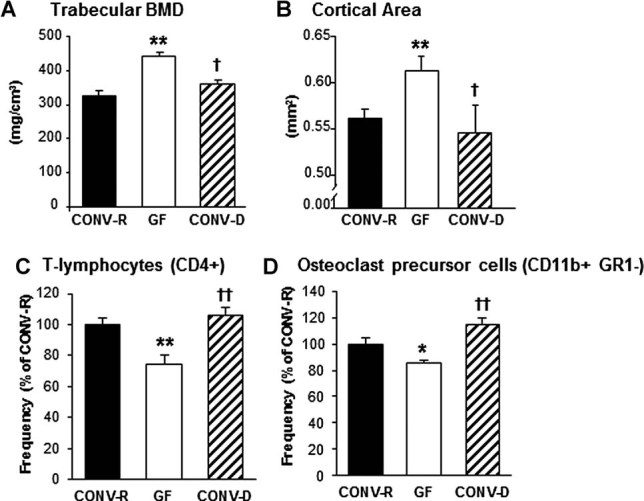
Colonization of GF mice with a normal gut microbiota normalizes bone mass and immune status in bone marrow. Analysis of bone and bone marrow in female 7-week-old GF and CONV-R mice and an extra control group consisting of mice that were born GF and then colonized with normal gut microbiota at 3 weeks of age (conventionalized; CONV-D). (*A*) Trabecular bone mineral density (BMD) measured by pQCT in the distal femur. (*B*) Cortical cross-sectional bone area measured by µCT in the diaphyseal region of femur. (*C*) Quantitative data for the frequency of T cells (CD4^+^) in the total bone marrow population; (*D*) Quantitative data for the frequency of CD11b^+^/Gr1^–^ cells (OCL precursor cells; see text for details) in the total bone marrow population. Values are given as mean ± SEM, *n* = 4 to 14 in each group. **p ≤ 0.01, GF versus CONV-R; †*p* ≤ 0.05, ††*p* ≤ 0.01, CONV-D versus GF, ANOVA followed by Tukey's post hoc test.

To exclude developmental factors and confirm that the absence of gut microbiota was directly responsible for the increased bone mass in GF mice, we colonized GF mice at weaning with a normal gut microbiota (conventionalized; CONV-D). Four weeks after colonization, CONV-D mice had significantly reduced trabecular vBMD compared with GF counterparts and were indistinguishable from CONV-R mice (Fig. 6*A*). Similar results were observed for cortical cross-sectional bone area (Fig. 6*B*). Colonization of GF mice did not significantly increase serum serotonin levels (GF 1.86 ± 0.23 µg/mL versus CONV-D 2.39 ± 0.16 µg/mL; NS) but led to a normalization of the frequency of CD4^+^ T cells and OCL precursor (CD11b^+^/Gr1^–^) cells in bone marrow (Fig. 6*C, D*).

## Discussion

In the current study, we show that absence of a gut microbiota leads to increased bone mass associated with reduced number of OCLs in trabecular bone and decreased frequency of CD4^+^ T cells and OCL precursor cells in bone marrow. The specificity of the effects of the gut microbiota on bone mass was demonstrated by colonizing mice that were born GF with a normal gut microbiota at 3 weeks of age, which led to a normalization of bone mass and the frequency of CD4^+^ T cells and CD11b^+^/GR 1^–^ OCL precursor cells in bone marrow. The increased bone mass in GF mice could not be explained by altered calcium metabolism.

The serum serotonin level was decreased in GF mice in combination with decreased expression of the rate-limiting enzyme for serotonin synthesis, Tph 1 in colon. Furthermore, the expression of the serotonin transporter SERT was increased, suggesting increased inactivation and breakdown of serotonin in colon of GF mice. Serum serotonin has earlier been reported to be 2.8-fold higher in CONV-R compared with GF mice.^44^ However, colonization of GF mice led to a normalization of bone mass but had no significant effect on serum serotonin, indicating that the high bone mass in GF mice was not primarily caused by altered serum serotonin levels.

The frequency of CD4^+^ T cells and OCL precursor cells were decreased in bone marrow in GF mice in combination with increased bone mass. GF mice have reduced number of CD4^+^ T cells in the spleen and fewer and smaller germinal centers within the spleen, suggesting that the gut microbiota is capable of shaping systemic immunity.^7^, ^8^ We extended these data by showing that also bone marrow from GF mice has reduced frequency of CD4^+^ T cells. CD4^+^ T cells can be broadly grouped, based on the type of cytokine they produce, into Th1 (IFNγ), Th2 (IL-4), and Th17 (IL-17). Of these, Th17 cells are osteoclastogenic and suggested to be responsible for the bone destruction phase of autoimmune arthritis.^18^ GF mice have been shown to have a Th2-skewed profile with increased production of IL-4 and decreased production of IFNγ from splenic CD4^+^ T cells in vitro.^8^ Interestingly, Th17 cell development is specifically affected by the gut microbiota, and GF mice are deficient in the production of IL-17 from CD4^+^ T cells, which is the signature cytokine of Th17 cells.^45^ Furthermore, GF mice display reduced Th17 cell numbers in the spleen and spinal cords.^7^, ^46^, ^47^ Whether Th17 cell numbers are also low in the bone marrow of GF mice remains to be determined. We suggest that the decrease in CD4^+^ T cells in bone marrow in GF mice is a consequence of the impact of the gut microbiota on the adaptive immune system, leading to fewer CD4^+^ cells recirculating in blood and secondary lymphoid tissue. Rheumatoid arthritis (RA) is an autoimmune disease characterized by inflammation of synovial joints with CD4^+^ T-cell infiltration in combination with an increased expression of inflammatory cytokines such as TNFα, leading to severe bone destruction mediated by OCLs.^48^ TNFα is one of the critical cytokines in the pathogenesis of RA, as shown by many gain- and loss-of-function genetic models, as well as by the clinical efficacy of anti-TNFα therapy.^49^, ^50^ Patients with psoriatic arthritis and mice with TNFα-induced arthritis have increased numbers of circulating OCL precursors, which correlate with systemically increased TNFα concentrations and are reduced by anti-TNFα therapy in association with clinical improvement.^51^–^53^ TNFα promotes osteoclastogenesis indirectly by stimulating RANKL expression by marrow stromal cells and osteoblasts and by direct stimulation of OCL precursors exposed to permissive levels of RANKL.^54^–^56^ Osteoclastogenesis can also be regulated by cytokines under physiological conditions, and T-cell–produced TNFα is thought to play a role in ovx-induced bone loss.^19^, ^57^ GF mice have low numbers of TNFα-producing CD4^+^ T cells in colon compared with CONV-R mice.^58^ In line with these data, we found decreased expression of TNFα in colon of GF mice. Furthermore, the expression of TNFα and IL-1 was decreased in bone marrow of GF mice probably as a consequence of the observed decrease in the frequency of CD4^+^ T cells in their bone marrow. Decreased levels of TNFα and IL-1 in bone marrow of GF mice would indicate decreased osteoclastogenesis, which is supported by the observed decrease in the frequency of CD11b^+^/GR1^–^ OCL precursor cells in bone marrow and decreased number of OCLs in trabecular bone of GF mice. Another inflammatory cytokine, interleukin-6 (IL-6) has been shown to stimulate OCL formation and function in vitro.^59^ IL-6 has complex effects in bone, and IL-6 knockout mice have only minor effects on bone but are protected against ovx-induced bone loss.^60^ IL-6 expression was decreased in bone but not in bone marrow in GF mice, suggesting that IL-6 is not responsible for the observed effects. Together these data indicate that the increased bone mass in GF mice depends on a decreased osteoclastogenesis probably as a consequence of a decrease in T-cell–produced TNFα. A limitation of the current study is the lack of direct mechanistic experiments assessing if the observed alterations in T cells and/or cytokines are involved in the high bone mass phenotype in GF mice. Whether any of the observed changes is critical for the effects of the gut microbiota on bone mass remains to be determined in future mechanistic studies.

Colonization of GF mice led to a normalization of bone mass and the frequency of CD4^+^ T cells and CD11b^+^/GR1^–^ OCL precursor cells in bone marrow, supporting the notion that the high bone mass in GF mice was dependent on influences of the gut microbiota on the immune system.

**Fig. 7:**
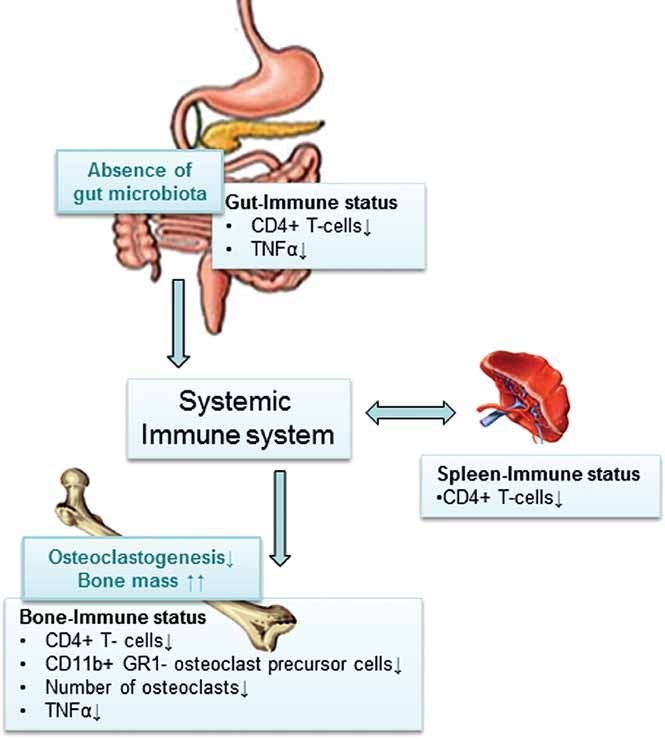
Proposed mechanism for how the gut microbiota regulates bone mass. Absence of gut microbiota leads to increased bone mass associated with reduced number of OCLs per bone surface, decreased frequency of CD4^+^ T cells and OCL precursor cells in bone marrow, and reduced expression of the osteolytic cytokine TNFα in bone. Earlier studies have shown that GF animals have immature mucosal immune systems with hypoplastic Peyer's patches containing few germinal centers and reduced number of IgA-producing plasma cells and lamina propria CD4^+^ T cells.^6^ Furthermore, GF mice have reduced number of CD4^+^ T cells in the spleen and fewer and smaller germinal centers within the spleen, suggesting that the gut microbiota is capable of shaping systemic immunity.^7^, ^8^ We propose that the increased bone mass is caused by fewer CD4^+^ cells recirculating in blood and secondary lymphoid tissue, resulting in a decreased frequency of CD4^+^ T cells in bone marrow associated with a decreased expression of inflammatory cytokines and less osteoclastogenesis in the absence of gut microbiota.

In summary, absence of gut microbiota leads to increased bone mass associated with reduced number of OCLs per bone surface, decreased frequency of CD4^+^ T cells and OCL precursor cells in bone marrow, and reduced expression of TNFα in bone and bone marrow. Colonization of GF mice with a normal gut microbiota led to a normalization of bone mass and immune status in bone marrow. We propose that the increased bone mass is caused by fewer CD4^+^ T cells recirculating in blood and secondary lymphoid tissue resulting in a decreased frequency of CD4^+^ T cells in bone marrow associated with a decreased expression of TNFα and frequency of OCL precursor cells, leading to decreased osteoclastogenesis in the absence of gut microbiota (Fig. 7). Further studies are required to evaluate the gut microbiota as a possible novel therapeutic target for osteoporosis.
