# Supplementary material for: The gut microbiota regulates bone mass in mice
Source: J Bone Miner Res. 2012 Jun;27(6):1357–67. doi: 10.1002/jbmr.1588 (PMC3415623; doi:10.1002/jbmr.1588)
Supplement: Supplementary file 1 [file jbmr0027-1357-SD1.docx]

**Supplemental Information**

**
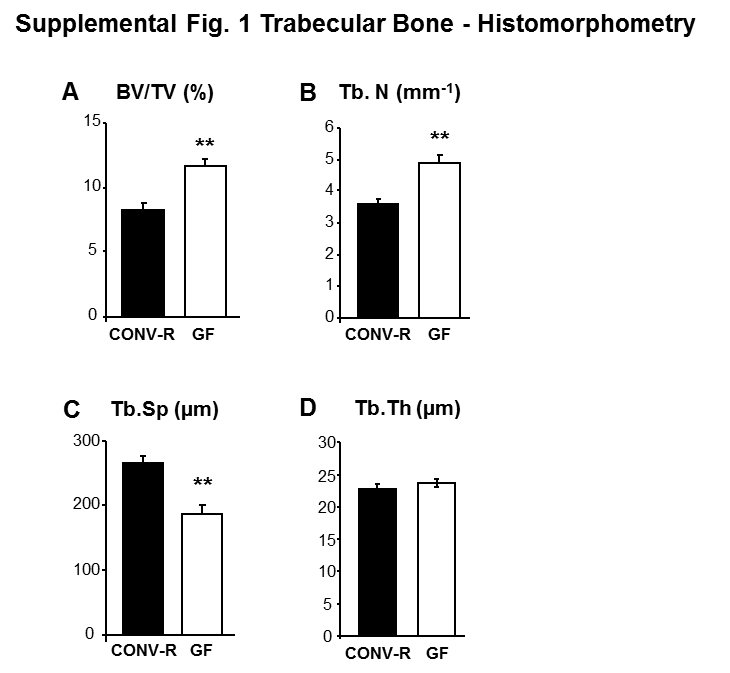
**

**Supplementary Fig. 1** Histomorphometric analysis of trabecular bone in the distal femur from 9-week-old GF and CONV-R female mice. (A) BV/TV (%), trabecular bone volume as a percentage of tissue volume; (B) Tb.N (mm^-1^), trabecular number; (C) Tb.Sp (µm), trabecular separation; (D) Tb.Th (µm), trabecular thickness. Values are given as mean±SEM, n=5-6. * p≤0.05, ** *P*≤0.01 versus CONV-R, student’s *t*-test.

**
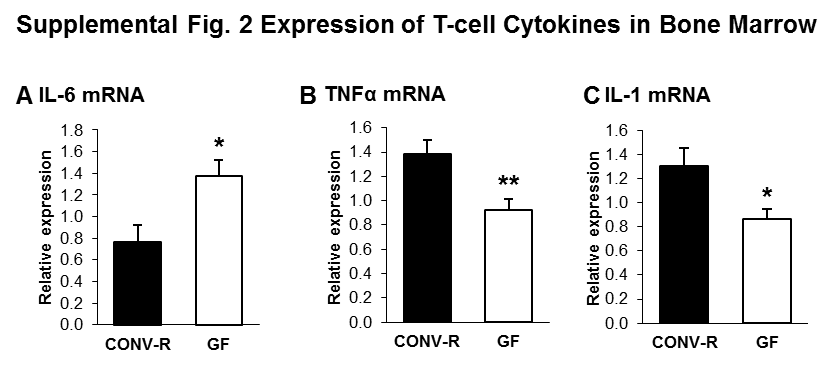
**

**Supplementary Fig. 2** QRT-PCR analysis of the expression of (A) Interleukin-6 (Il-6), (B) Tumor Necrosis Factor alpha (TNFα) and Interleukin-1 (IL-1) in bone marrow from GF and CONV-R female mice. Values are given as mean±SEM, n=7-10. * p≤0.05, ** *P*≤0.01 versus CONV-R, student’s *t*-test.

**Supplemental Table.**

|  | **CONV-R** | **GF** |
| --- | --- | --- |
| **Serum Ca^2+^ (mg/dl)** | 8.7±0.2 | 9.0±0.2 |
| **Serum PTH (pg/ml)** | 41.8±22.3 | 41.4±15.9 |
| **Serum 25(OH)D_3_ (nmol/l)** | 78.8±2.8 | 76.2±1.5 |

Calcium homeostasis measured in 9-week-old GF and CONV-R female mice. Values are given as mean±SEM, n=5-6 in each group.
